# Optimal Timing of Tracheostomy in the Setting of COVID-19 and Associated Pneumothorax

**DOI:** 10.7759/cureus.55479

**Published:** 2024-03-04

**Authors:** Matthew L Zweerink, Hilla I Sang, Adam K Durrani, Khaled Zreik

**Affiliations:** 1 General Surgery, University of North Dakota School of Medicine and Health Sciences, Grand Forks, USA; 2 Research Design and Biostatistics Core, Sanford Health, Fargo, USA; 3 School of Medicine, Royal College of Surgeons in Ireland, Dublin, IRL; 4 Surgical Critical Care, Sanford Medical Center, Fargo, USA; 5 Surgical Critical Care, University of North Dakota School of Medicine and Health Sciences, Grand Forks, USA

**Keywords:** covid-19, pneumothorax (ptx), critical care outcomes, critical care, tracheostomy placement, tracheostomy

## Abstract

Introduction

At the beginning of the 2020 pandemic, no criteria were in place regarding the timing of tracheostomy placement in intubated COVID-19 patients, nor were there any data pertaining to pneumothorax incidence in this population. This study examines the timing of tracheostomy placement and its correlation with patient outcomes, along with pneumothorax incidence in COVID-19 patients who underwent a tracheostomy.

Methods

We performed a multi-institutional retrospective study of intubated COVID-19 patients admitted to intensive care units (ICUs) in North and South Dakota between April 2020 and December 2020. The timing of the tracheostomy was assessed, with primary outcomes being mortality, successful ventilator weaning, discharge to a long-term care facility, and overall length of stay. Patients were grouped by age, gender, ethnicity, and comorbidities. Pre- and post-tracheostomy pneumothorax was extracted from this dataset.

Results

We identified 85 patients who were intubated with COVID-19 and underwent a tracheostomy. The timing of tracheostomy varied widely, ranging from five to 53 days with an average time to tracheostomy being 17.3 days. Thirty-four of the patients expired, 32 patients were discharged to a long-term care hospital (LTCH), and 11 patients were discharged to an inpatient rehabilitation facility. Only three patients were discharged home. Regression analysis did not reveal statistically significant differences between patients who survived (N = 51) and patients who expired (N = 34) for almost all variables analyzed. Sixteen of the 85 patients were diagnosed with pneumothorax during their hospital stay. Half of these patients were diagnosed after a tracheostomy was placed.

Conclusion

This study did not demonstrate statistically significant differences in overall mortality or incidence of pneumothorax when it pertains to the timing of placement of tracheostomy. Variation in mortality was identified, in which younger patients were more likely to survive than older patients, a finding that was echoed in other studies. Considering this evidence, we cannot conclude that an association between the timing of tracheostomy and mortality from COVID-19; therefore, tracheostomy in the setting of COVID-19 can be performed at the provider's discretion.

## Introduction

During the COVID-19 pandemic’s onset, the nation’s intensive care units (ICUs) were overwhelmed with critically ill patients who were unable to wean from mechanical ventilators. Reports of mortality rates higher than 50% among patients who required intubation, and in some settings as high as 97% [1], raised concerns about the efficacy of intubation as a safe intervention for COVID-19 patients. An institutional protocol regarding tracheostomy timing for ventilated COVID-19 patients was implemented. The aerosolizing procedure required multiple hospital staff members to complete, which raised additional concerns due to a shortage of personal protective equipment (PPE) [2].

While no official guidelines currently exist, the general consensus among critical care providers is to perform tracheostomy prior to 14 days of intubation [3]. This practice prevents complications associated with orotracheal intubation, such as ulcerations, and provides increased patient comfort. In addition, increased positive end-expiratory pressure (PEEP) needed to treat the profound pulmonary damage caused by COVID-19 carries an increased risk of pneumothorax [4].

The benefits of tracheostomy include decreased intrinsic PEEP, which occurs because intrinsic PEEP, also known as auto-PEEP or trapped air, happens when air remains in the lungs at the end of expiration due to incomplete lung emptying. A tracheostomy reduces airway resistance and dead space compared to endotracheal tubes, facilitating more complete exhalation and reducing the buildup of intrinsic PEEP. This decreases the risk of barotrauma (lung injury due to overdistension) and improves overall lung mechanics. Placement of a tracheostomy also decreases the work of breathing by reducing airway resistance and intrinsic PEEP. A tracheostomy can lower the effort needed to breathe, especially during spontaneous breathing trials or when the patient is partially ventilated. This can lead to less respiratory muscle fatigue and better oxygenation [5-6].

Advantages also include improved patient comfort, decreased sedation requirements, and earlier mobilization; tracheostomy is more comfortable and less stimulating than an endotracheal tube, and patients often require less sedation. This reduction in sedation levels allows for earlier cognitive and physical assessment and can expedite the process of weaning off mechanical ventilation.

With decreased sedation and increased comfort, patients are often able to participate in physical therapy sooner. Early mobilization is critical for preventing muscle weakness and deconditioning, which are common complications of prolonged bed rest and mechanical ventilation [7-8]. In addition, tracheostomy has proven beneficial in patients expected to have prolonged mechanical ventilation, such as COVID-19 patients, due to improved weaning rates and reduced complications that contribute to better overall outcomes and potentially lower mortality rates, particularly in patient populations with prolonged ventilation needs [9]. 

Given the unprecedented circumstances of the COVID-19 pandemic, this study has two aims: First, to explore the impact of tracheostomy timing on patient outcomes, we are hypothesizing that an earlier tracheostomy will lead to early mobilization of patients, decreased sedation, and improved mortality. Second, to investigate whether there is a correlation between the placement of a tracheostomy and the incidence of pneumothorax following the procedure, we are hypothesizing that the placement of a tracheostomy might lead to a decreased incidence of pneumothorax as it will likely decrease the PEEP requirements during mechanical ventilation. 

This article was previously presented as an abstract and oral presentation at the 2022 Southwestern Surgical Congress Meeting on April 27, 2022.

## Materials and methods

Study design

We conducted a retrospective observational cross-sectional study. We collected data from electronic medical records (EMRs) to identify outcomes of patients with COVID-19 pneumonia who underwent tracheostomy (C19PT) in Sanford Health, a single trauma center in the upper Midwest region of the United States.

Settings

Data for the study were collected in 2021 on patients admitted at Sanford Health in 2020. As the largest rural healthcare provider in the US, Sanford Health was uniquely positioned to identify and quantify the relationship between C19PT and clinical outcomes. Given its vast geographic coverage, and because many local and small hospitals closed during the pandemic throughout the upper Midwest region, many patients were diverted to the large Sanford Health facilities for treatment. Therefore, Sanford Health cared for a population that represented the experience of COVID-19 in the region. 

Participants

The study participants included all patients 18 years or older who underwent tracheostomy for COVID-19 pneumonia who were admitted between March 2020 and December 2020 to Sanford Health Medical Center in Fargo, North Dakota.

Variables

The main outcome variable was in-hospital mortality. Secondary outcomes were days on the ventilator, discharge disposition, and overall length of stay. Timing of tracheostomy was measured in days from two separate time points: (a) from day of COVID-19 diagnosis and (b) from day 1 of ventilation.

Data sources/measurements

Data were collected from each patient’s EMR. Variables included patient demographic characteristics (age, sex, and race), preexisting conditions (coronary heart disease, hypertension, diabetes, congestive heart failure, stroke, and BMI), date of COVID-19 diagnosis, date of admission, date of tracheostomy, pneumothorax diagnosis, and discharge disposition. Given the relatively homogenous population in the region, the patient's race was reported from the patient's first reported race and was grouped as either White, African American or Black, Indigenous American, and all other races.

Statistical methods

Chi-squared and Kruskal-Wallis rank-sum tests were conducted to examine the relationship between mortality and the various interventions. A multivariate logistic regression model was fitted to predict mortality in the study population. The significance level was set at α=0.05. Statistical analysis was conducted in R. The study was granted exempt status by Sanford Health Institutional Review Board (approval no. STUDY00002303). Procedures were followed in accordance with the Helsinki Declaration of 1975.

## Results

Patient characteristics and comorbidities

Of the 85 patients in the study, 40% (N = 34) were female, about one-quarter (23.5%, N = 20) were Indigenous American, and 58.8% were White (N = 50) (Table 1). The mean BMI was 32.7 (standard deviation (SD) = 7.5, interquartile range (IQR) = 27.24-37.23). The most frequent comorbidity was stroke, affecting 88.2% of the patients (N = 75), followed by coronary artery disease (CAD) (87.1%, N = 74). No statistically significant differences in characteristics, comorbidities, or length of stay were observed between patients who survived (N = 51) and patients who expired (N = 34).

**Table 1:** Patient characteristics and comorbidities by mortality ^1^Kruskal-Wallis rank sum test, ^2^Pearson’s Chi-squared test

	Mortality (N = 34)	Survival (N = 51)	Total (N = 85)	p-value
Age				0.129^1^
Median (Q1, Q3)	64.0 (54.5, 72.5)	62.0 (52.5, 67.5)	63.0 (53.0, 70.0)	
Gender				0.857^2^
Female	14 (41.2%)	20 (39.2%)	34 (40.0%)	
Male	20 (58.8%)	31 (60.8%)	51 (60.0%)	
Race				0.609^2^
AI/AN	10 (29.4%)	10 (19.6%)	20 (23.5%)	
African American/Black	2 (5.9%)	2 (3.9%)	4 (4.7%)	
Caucasian/White	19 (55.9%)	31 (60.8%)	50 (58.8%)	
All other races	3 (8.8%)	8 (15.7%)	11 (12.9%)	
BMI				0.200^1^
Median (Q1, Q3)	32.9 (28.6, 39.6)	30.2 (27.02, 35.8)	31.8 (27.2, 37.2)	
Coronary artery disease				0.692^2^
No	29 (85.3%)	45 (88.2%)	74 (87.1%)	
Yes	5 (14.7%)	6 (11.8%)	11 (12.9%)	
Diabetes				0.929^2^
No	19 (55.9%)	28 (54.9%)	47 (55.3%)	
Yes	15 (44.1%)	23 (45.1%)	38 (44.7%)	
Hypertension				0.177^2^
No	17 (50.0%)	18 (35.3%)	35 (41.2%)	
Yes	17 (50.0%)	33 (64.7%)	50 (58.8%)	
Congestive heart failure				0.899^2^
No	29 (85.3%)	44 (86.3%)	73 (85.9%)	
Yes	5 (14.7%)	7 (13.7%)	12 (14.1%)	
Stroke				0.492^2^
No	31 (91.2%)	44 (86.3%)	75 (88.2%)	
Yes	3 (8.8%)	7 (13.7%)	10 (11.8%)	
Length of stay (days)				0.118^1^
Median (Q1, Q3)	35.0 (25.7, 47.5)	43.6 (29.2, 58.7)	38.9 (28.9, 55.7)	

Ventilation

The number of days on the ventilator ranged from three to 173 (M = 39.05, SD = 25.123) (Table 2). Most ventilated patients (67.9%, N = 36) had a positive end-expiratory pressure (PEEP) value of eight at the time of tracheostomy; PEEP values at the time of tracheostomy were available for only 55 of the 85 patients. A Kruskal-Wallis rank-sum test found that patients who expired had statistically significantly (p = 0.017) lower number of days on ventilator (33.7 ± 23.3 days) than patients who survived (42.5 ± 25.8 days).

**Table 2:** Ventilation by mortality outcome ^1^Kruskal-Wallis rank-sum test, ^2^Pearson’s Chi-squared test PEEP: positive end-expiratory pressure

	Mortality (N = 34)	Survival (N = 51)	Total (N = 85)	p-value
Days vented				0.017^1^
Median (Q1, Q3)	26.5 (19.25, 37.750)	38.0 (28.5, 52.0)	34.0 (23.0, 48.0)	
Days from COVID diagnosis to tracheostomy				0.733^1^
Median (Q1, Q3)	24.5 (16.25, 36.0)	22.0 (16.0, 29.5)	22.0 (16.0, 31.0)	
Days from vent start to tracheostomy				0.526^1^
Median (Q1, Q3)	15.0 (11.25, 21.0)	16.0 (13.0, 19.0)	16.0 (12.0, 21.0)	
PEEP value at time of tracheostomy (n, %)				0.363^2^
6	0 (0.0%)	2 (5.3%)	2 (3.8%)	
8	8 (53.3%)	28 (73.7%)	36 (67.9%)	
10	2 (13.3%)	2 (5.3%)	4 (7.5%)	
12	2 (13.3%)	3 (7.9%)	5 (9.4%)	
14	2 (13.3%)	2 (5.3%)	4 (7.5%)	
16	0 (0.0%)	1 (2.6%)	1 (1.9%)	
18	1 (6.7%)	0 (0.0%)	1 (1.9%)	

Discharge disposition

Thirty-four (40.0%) of the patients in the study expired, 32 (37.6%) patients were discharged to a long-term hospital (LTCH), and 11 (12.9%) patients were discharged to an inpatient rehabilitation facility. Only three patients were discharged to self-care at home (Table 3). 

**Table 3:** Discharge disposition

	Overall (N = 85)
Discharge disposition	
Expired	34 (40.0%)
Home under the care of an organized home health service organization	1 (1.2%)
Home, self-care	3 (3.5%)
Hospice – home	1 (1.2%)
Hospital-based Medicare-approved swing bed	1 (1.2%)
Inpatient rehab facility (IRF) including rehab DPU of a hospital	11 (12.9%)
Medicare-certified long-term care hospital (LTCH)	32 (37.6%)
Short-term general hospital for inpatient care	1 (1.2%)
Skilled nursing facility (SNF) w/ Medicare certification	1 (1.2%)

Logistic regression modeling

Results from a logistic regression analysis suggest that none of the variables, other than patient age, predict mortality in a statistically significant level (p = 0.05) (Tables 4, 5, 6).

**Table 4:** Logistic regression of patient demographics by mortality ^1^OR = odds ratio, CI = confidence interval

Characteristic	OR^1^	95% CI^1^	p-value
Age	0.96	0.93, 1.00	0.05
Gender			
Female	—	—	
Male	1.02	0.40, 2.58	0.96
Race			
AI/AN	—	—	
African American/Black	1.18	0.12, 11.9	0.88
Caucasian/White	2.25	0.73, 7.25	0.16
All other races	4.24	0.84, 26.5	0.095

**Table 5:** Logistic regression of patient mortality by comorbidities ^1^OR = odds ratio, CI = confidence interval BMI: body mass index, CAD: coronary artery disease, HTN: hypertension, CHF: congestive heart failure

Characteristic	OR^1^	95% CI^1^	p-value
BMI	0.95	0.89, 1.01	0.13
CAD	0.51	0.12, 2.11	0.34
Diabetes	0.95	0.35, 2.51	0.91
HTN	2.36	0.85, 6.83	0.1
CHF	1.04	0.29, 4.01	0.96
Stroke	1.21	0.28, 6.27	0.8

**Table 6:** Logistic regression of patient mortality by count of days ^1^OR = odds ratio, CI = confidence interval

Characteristic	OR^1^	95% CI^1^	p-value
Days vented	1.02	1.00, 1.05	0.1
Days from COVID Dx to tracheostomy	0.98	0.94, 1.00	0.24
Days from vent start to tracheostomy	1	0.95, 1.07	0.92

Pneumothorax

Sixteen of the 85 patients (18.8%) were diagnosed with pneumothorax during their hospital stay. Half of those patients (N = 8) received their pneumothorax diagnosis after tracheostomy placement. Mortality occurred in five patients (62.5%) whose pneumothorax occurred after tracheostomy, while patients whose pneumothorax preceded tracheostomy placement died in 50% of the cases (N = 4). No statistical significance was observed.

## Discussion

At the beginning of the 2020 COVID-19 pandemic, ICUs across the US were faced with the challenge of dealing with multiple patients on ventilators who could not be mobilized outside of the ICU [10]. The placement of a tracheostomy on patients who are stable clinically, but are not ready to be extubated, can facilitate their transfer outside the ICU [11]. 

One of the barriers identified in mobilizing COVID-19 ICU patients was a delay in the placement of a tracheostomy tube [11]. Delay of tracheostomy placement was due to various factors, including the provider's fear of contracting the virus, the high level of PEEP these patients were exposed to, and the high mortality at the beginning of the pandemic associated with intubated COVID-19 patients. However, an anonymous international survey examining factors associated with COVID-19 infection found that performing a tracheostomy was not associated with COVID-19 infection, suggesting that tracheostomies can be safely performed in infected patients with appropriate precautions [12]. 

Various institutions at the time established protocols to address the timing and indication of tracheostomy placement in this subset of intubated patients [13]. These protocols were mostly extrapolated from experience with non-COVID intubated patients who were primarily diagnosed with pneumonia. There were variations of protocols. For example, Chao et al. originally recommended deferring tracheostomy beyond 21 days of intubation and recommended open surgical tracheostomy over percutaneous dilatational tracheostomy [14]. Conversely, a multicenter study conducted in 2021 by Mahmood et al. has shown that early (≤14 days), percutaneous tracheostomy was associated with improved outcomes compared to surgical tracheostomy in a multi-institutional series of ventilated patients with COVID-19 [15].

Early studies have shown that tracheostomy is safe in patients who had prolonged intubation [1]. However, at the time when this study was being conducted, no studies were published pertaining to the timing of tracheostomy placement in COVID-19 patients [13] and measuring the relationship between tracheostomy and pneumothorax incidence. 

This study is a retrospective study analyzing the timing of tracheostomy placement and its association with patient outcomes and pneumothorax incidence. We found that post tracheostomy, there were no statistically significant differences among patient groups for almost all variables analyzed. Notably, younger patients demonstrated increased survival (p = 0.05); this finding was also observed by a large study that showed that greater age, among other factors, was an independent risk factor for death following hospitalization for COVID-19 [16]. Hence, we stipulate that the similar finding in our study is unlikely to be related to tracheostomy specifically.

A multicenter study conducted for the National Library of Medicine and published after our study was concluded showed similar findings. With a broad range of tracheostomy timing, there was no difference in overall survival. They did demonstrate decreased time spent in the ICU [11]. This variable was not examined in our study. These findings are consistent with other studies pertaining to tracheostomies performed on non-COVID patients that showed no superiority of early vs late tracheostomies [17].

The incidence of pneumothorax was identified as another outcome. We did not find an association between tracheostomy and pneumothorax incidence. This is most likely due to the fact that we were unable to demonstrate that PEEP could be decreased following the procedure. A recent study has shown that air leak syndromes (pneumothorax and pneumomediastinum) were common findings in COVID-19 patients following placement of a tracheostomy. In this study, six out of 136 patients enrolled developed pneumothorax two to three days after tracheostomy, and 10 out of 136 patients had pneumothorax prior to placement of the tracheostomy, but no direct correlations have been established [18].

The findings of this study should be examined with the following limitations. First, this study utilized information from patient electronic medical charts. The authors had no control over the quality and completeness of the data. Due to the chaotic time in which the data were recorded, some data, like PEEP values, were missing for almost one-third of the patients. However, the authors included all available patient data rather than a sample, which resulted in findings that best represent the challenges and outcomes of the COVID-19 pandemic. Second, the retrospective nature of this study means that there was no preselection or screening of study participants, nor was there a predetermined division of study participants into treatment and control groups. While this study does not provide the highest level of evidence of the relationship among COVID-19, tracheostomy, and patient outcomes, the strength of this study lies within the representation of the upper Midwest population, a population that is vastly under-studied and under-represented in research literature, and its experience of COVID-19.

## Conclusions

This study did not demonstrate statistically significant differences in overall mortality or incidence of pneumothorax when it pertains to the timing of placement of tracheostomy. Variation in mortality was identified, in which younger patients were more likely to survive than older patients, a finding that was echoed in other studies. Considering this evidence, we cannot conclude that an association between the timing of tracheostomy and mortality from COVID-19; therefore, tracheostomy in the setting of COVID-19 can be performed at the provider's discretion.
